# NeuroLex.org: an online framework for neuroscience knowledge

**DOI:** 10.3389/fninf.2013.00018

**Published:** 2013-08-30

**Authors:** Stephen D. Larson, Maryann E. Martone

**Affiliations:** Department of Neurosciences, University of California San DiegoLa Jolla, CA, USA

**Keywords:** wiki, knowledge management, neuroanatomy, ontology, semantics

## Abstract

The ability to transmit, organize, and query information digitally has brought with it the challenge of how to best use this power to facilitate scientific inquiry. Today, few information systems are able to provide detailed answers to complex questions about neuroscience that account for multiple spatial scales, and which cross the boundaries of diverse parts of the nervous system such as molecules, cellular parts, cells, circuits, systems and tissues. As a result, investigators still primarily seek answers to their questions in an increasingly densely populated collection of articles in the literature, each of which must be digested individually. If it were easier to search a knowledge base that was structured to answer neuroscience questions, such a system would enable questions to be answered in seconds that would otherwise require hours of literature review. In this article, we describe NeuroLex.org, a wiki-based website and knowledge management system. Its goal is to bring neurobiological knowledge into a framework that allows neuroscientists to review the concepts of neuroscience, with an emphasis on multiscale descriptions of the parts of nervous systems, aggregate their understanding with that of other scientists, link them to data sources and descriptions of important concepts in neuroscience, and expose parts that are still controversial or missing. To date, the site is tracking ~25,000 unique neuroanatomical parts and concepts in neurobiology spanning experimental techniques, behavioral paradigms, anatomical nomenclature, genes, proteins and molecules. Here we show how the structuring of information about these anatomical parts in the nervous system can be reused to answer multiple neuroscience questions, such as displaying all known GABAergic neurons aggregated in NeuroLex or displaying all brain regions that are known within NeuroLex to send axons into the cerebellar cortex.

## Introduction

There has been a great deal of work in the study of “knowledge representation” in computer science (Davis et al., 1993). Knowledge representation concerns itself with how to capture the meaning of statements in machine processable forms. Examples of statements of interest to neuroscience include “mitochondria are part of neurons” and “Purkinje cells are located in the cerebellar cortex.” In these examples, “mitochondria,” “Purkinje cells,” neurons and cerebellar cortex are the entities, “part of” and “located in” are properties (sometimes referred to as relations or relationships). By splitting knowledge into these atoms, computational systems can better analyze the relationships expressed within them. This makes individual statements available for search, query, and reuse into other information systems, which is still currently difficult with unstructured prose. When done over a large knowledge base, this approach enables computational systems to keep large amounts of complex information well-organized and easily accessible, enables search across distributed databases and allows data-minded scientists to rapidly pose and answer questions about existing knowledge via automated logical deductions (Martone et al., 2004; Larson and Martone, 2009). Computer science is increasingly producing tools to find patterns in large corpuses of data that have been structured this way (Abu-Mostafa et al., 2012). However, a well-structured knowledge base that has been comprehensively populated and has built up significant consensus from the neuroscience community is far from completion.

Producing structured knowledge for the purposes of organizing, indexing and searching information has a long history in the biosciences. Well before the availability of large electronic data repositories or the Internet, systematized nomenclatures such as the Systematized Nomenclature of Medicine, Clinical Terms (SNOMED CT; Cornet and de Keizer, 2008) and the Unified Medical Language System; UMLS; Lindberg et al., 1993) were in use. As the number and depth of electronically accessible, potentially networked data resources began to explode, the drive to develop machine-processable models of human knowledge in multiple disciplines to organize and integrate these data has similarly accelerated (Noy et al., 2009). These developments brought about an increased usage of “ontologies,” intended to enable knowledge representation to go beyond what was currently possible with databases.

The word “ontology” takes its meaning from the branch of philosophy concerned with categories of entities that exist in the world, and the relationships of similarity and difference between them. The more specialized sense of ontology has been applied by computer and information science, as computer systems have provided a means to use digital logic to enforce constraints of rigor on descriptions of entities (Gruber, 1993; Antoniou and Harmelen, 2009). Why work with ontologies instead of relying on databases? While databases are extremely powerful means of capturing and organizing data, one of the challenges of their usage for open-ended discovery is that the relationships between data types may change rapidly as new information becomes available. Due to the fact that database columns are separate data objects from rows, there is no explicit, strongly-typed^1^ relationship between data entities as there are in schemes such as the Resource Description Framework (RDF) (Spyns et al., 2002). Because of the practical problems using standard relational databases, some researchers organizing biological information turned to entity-attribute-value databases (Miller et al., 2005) or ontologies. For a more detailed review of ontologies and their applications in the neurosciences, please see Larson and Martone (2009).

### The neuroscience information framework

Based on the recognition of a lack of a consistent semantic framework for the neurosciences, the National Institutes of Health's Blueprint for Neuroscience Research project created The Neuroscience Information Framework (NIF; http://neuinfo.org; Gardner et al., 2008) in 2005. The project was conceived both to provide a current inventory of resources (tools, materials, data) relevant for neuroscience and to provide the means by which such resources could be effectively searched. Thus, NIF was asked with providing the necessary semantic framework for constructing and annotating such resources to promote their discovery and utilization. The NIF has been available in production since Fall of 2008. It is supported by an expansive lexicon and ontology, built through the synthesis of open access community ontologies, covering the broad domains of neuroscience and an infrastructure for bringing together diverse data sets into a single portal (Bug et al., 2008; Imam et al., 2012). The current NIF lists over 5500 individual resources of relevance to neuroscience and its virtual data federation brings together millions of records from independently maintained databases.

NIF builds upon several foundational efforts to provide a semantically-enhanced framework for searching across distributed data. The Brain Info system (Bowden and Dubach, 2003) incorporated the NeuroNames ontology to allow researchers to search for information on brain structures. The Biomedical Informatics Resource Network produced a comprehensive cross-disciplinary ontology for the neurosciences, extracting concepts (individual entries in an ontology that have a name and a series of properties) from UMLS, NeuroNames and other community ontologies. This ontology, called BIRNLex, evolved into the Neuroscience Information Framework (http://neuinfo.org) Standard ontology (NIFSTD); Bug et al., 2008). NIFSTD covers behavioral activity, behavioral paradigms, brain regions, cells, diseases, molecules, nervous system function, subcellular components, information resources, resource types, and qualities. The NIFSTD neuroanatomy is largely derived from the NeuroNames hierarchy.

### Challenges and motivation

In assembling the NIFSTD, NIF was able to take advantage of the many community ontologies created to cover its major domain areas. At the time, with the exception of NeuroNames, few of these ontologies specifically focused on the nervous system and few contained the type of specialized knowledge required by the NIF to effectively integrate across the hundreds of information sources created by neuroscientists. Neuroscience represents a challenge for ontology. First, the domain is a poor candidate because the domain of all entities relevant to neurobiological function is extremely large, highly fragmented into separate subdisciplines, and riddled with lack of consensus (Shirky, 2005).

The breadth of neuroscience also means that no single individual or group can cover the multiplicity of domains, structural and temporal scales required for both broad and deep coverage of neuroscience. Ontologies deal in human knowledge and thus require humans to provide the initial and final vetting of any knowledge base. The need for humans to structure knowledge limits the rate that knowledge can be ingested into machine-processable systems. While efforts such as Textpresso (Müller et al., 2004, 2008) have made progress applying automated text-mining techniques to this problem, a completely automated solution has not yet emerged. As a result, most projects hire dedicated curators and in some case ontologists for the task of knowledge engineering, data entry and data processing within their specific information systems. In short, curation and knowledge engineering in the biosciences is still very manual, highly technical, and therefore costly.

A third challenge is that the tools used to create and maintain biomedical ontologies require a lot of specialized knowledge and have not been inherently collaborative, though some are moving that direction. The most popular and functional ontology editors, such as Protégé (Rubin et al., 2007) or OBO-edit (Day-Richter et al., 2007), were originally designed for single user interaction. Only recently have collaborative editing tools begun to emerge with full support for ontologies (Tudorache et al., 2010, 2011). Moreover, Protégé and OBO-edit were designed as stand-alone applications not suitable for display on the World Wide Web. Only with the emergence of the BioPortal (Noy et al., 2009) and Web Protégé (Tudorache et al., 2011) have web-accessible interfaces to ontologies been made available.

A final challenge is the disconnect between the communities concerned with making primary observations of biology knowledge and those concerned with creating machine-processable representations of that knowledge. Following directly from the high costs of describing biological observations in machine processable forms, both in terms of skills and technologies, it has historically been difficult for working scientists to derive value from ontologies. Ontologies have been difficult to find, difficult to examine, and even more difficult for domain scientists to correct or enhance. As structuring knowledge for machines is akin to writing code, many classes and relationships in ontologies make little inherent sense to a domain scientist, yet most ontology editing tools expose this level of complexity. For these reasons, the barrier to entry is high for a biological scientist engaged in the observation of living systems—also a highly technical endeavor—to cross over into the area of biomedical ontologies and easily make useful contributions. This high barrier to entry has provided a disincentive that has kept the communities of neuroscience apart from the community of biological ontology, preventing the necessary crosstalk between these disciplines.

To address these issues, NIF looked to emerging technologies that might offer alternative ways to collaborate in producing and editing structured knowledge that would broaden the possible community of contributors to and consumers of this knowledge. Based on the success of community knowledge-building projects such as Wikipedia (http://wikipedia.org), we turned to a semantic wiki platform to create an interface to the NIFSTD where:

- Every entry is a web page
- Every entry can be edited individually
- Editing an entry was easy for motivated domain experts
- Linking entries was straightforward

In this article, we describe NeuroLex.org, a semantic wiki-based website and knowledge management system, the goal of which is to bring the complex frontier of knowledge within neurobiology into a framework that allows neuroscientists to review the anatomical features and principal concepts of neuroscience, link these features and concepts to resources, aggregate their personal understanding with that other scientists, and expose features and concepts that are still controversial or missing. To date, the site is tracking ~25,000 unique entities in neurobiology spanning experimental techniques, behavioral paradigms, anatomical nomenclature, genes, proteins and molecules. We describe the design and population of the Neurolex, and we show examples of how Neurolex can be used to answer questions about the nervous system that are currently difficult without extensive mining of the literature.

## Methods

NeuroLex is built using the Semantic MediaWiki platform v1.15.1 (Krotzsch et al., 2006). Semantic MediaWiki is an extension on the MediaWiki software that is the foundation of Wikipedia and supports millions of users a day. Semantic MediaWiki extends MediaWiki by allowing users to formalize knowledge through the use of special tags within wiki text. This means that a page in Semantic MediaWiki can be marked up to reveal knowledge within it in a structured way. For example, a wiki page about a neuron can indicate that its neurotransmitter is GABA and its soma is located in the hippocampus. This feature allows a wiki page to serve essentially as a database record for the topic that it covers, going beyond a simple text entry and allowing software to be written to analyze and synthesize content across pages.

NeuroLex was originally populated with entries from the main modules of NIFSTD, which tended to focus on multiscale structural anatomy. The initial update process, written with custom scripts using the PyWikipedia bot python framework, created one wiki page from each ontological class in the ontology. The import retained parent-child (super-category/sub-category) and part of relationships between classes.

To provide some basic structure to the Wiki suitable for neuroscience content, we utilized the Semantic Forms extension. This extension allows construction of customizable forms where range and domain can be established for each field. Thus, each field can be filled in via autocomplete by a subset of the terms within the wiki customized to each field. Because of this, any new content added to the wiki as a category page immediately becomes available for auto-completion. Additionally, the forms shield users from relying on sometimes arcane conventions of wiki text when editing or adding to a page.

**Figure 1:**
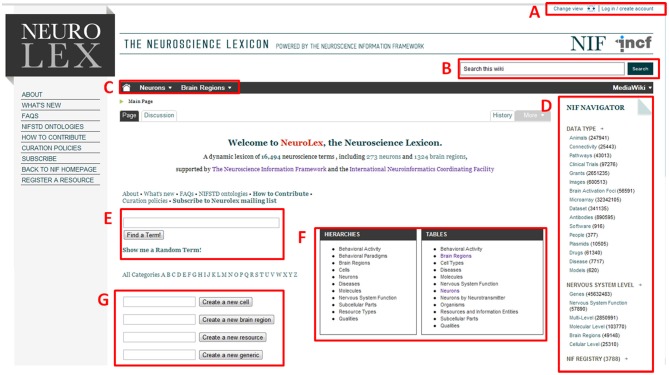
**Landing page for NeuroLex.org**. Several features are highlighted. **(A)** Login/user management controls. **(B)** Global site search bar. **(C)** Quick navigation to neuron or brain region information. **(D)** NIF Navigator, connecting the Neuroscience Information Framework b's federated resources to each NeuroLex page. **(E)** Global site search bar. **(F)** Quick navigation to hierarchies or tables containing detailed information about diverse entities in Neuroscience. **(G)** Quick creation forms for cells, brain regions, resources, and generic page contents.

**Figure 2:**
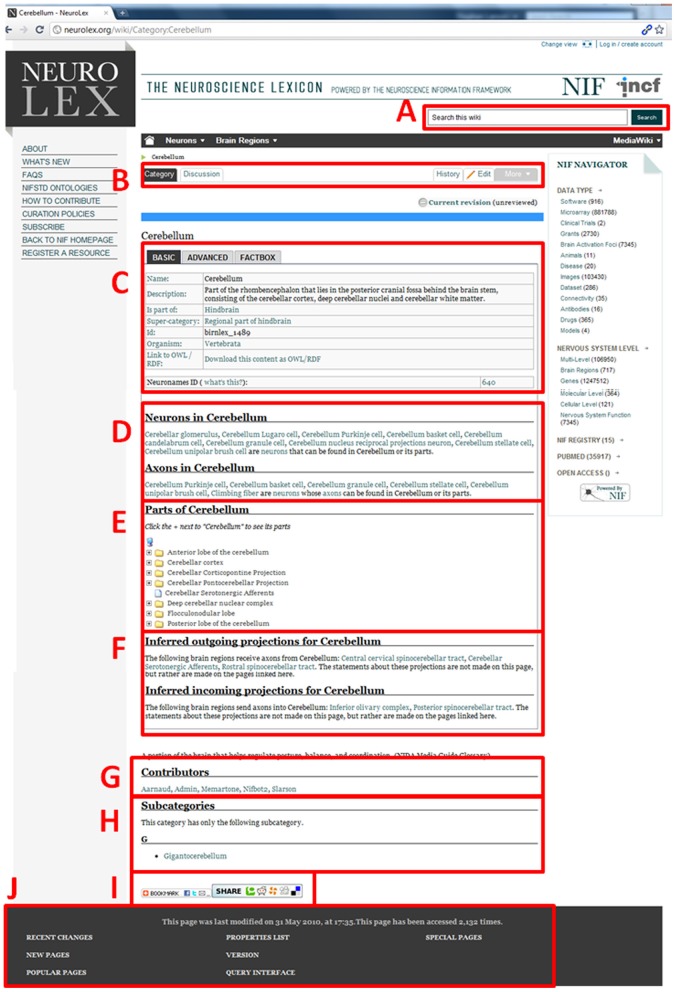
**Example category page for the concept entry ”Cerebellum” (A) Global site search bar. (B)** Wiki controls for this page, including link to a discussion page, page edit history, and edit controls for this page. **(C)** Basic facts for this entry, including text description, super category and more. Tabbed interface also contains additional advanced facts. **(D)** Advanced auto-generated report for neurons whose somas or axons are located in the Cerebellum. **(E)** Advanced auto-generated report of other brain regions that are listed as being a part of the cerebellum. **(F)** Advanced auto-generated report of outgoing and incoming projections for the cerebellum **(G)** List of users that have made edits to this page. **(H)** List of subcategories for this entry, i.e. concepts that are more specific than this current concept. **(I)** A widget that allows users to share this page with their social networks. **(J)** A global footer that contains last modified information, as well as site-wide information like recent changes, a list of new pages, special reports, and version information.

After installing Semantic Forms, the properties, templates, and forms were designed and created to assist users in providing structured information. We created a basic form that provides basic lexical information about the category, e.g., definition, synonyms, abbreviations, identifier, and basic relationships to other entities, e.g., is part of, super-category, related to. In the “detail” and “advanced” tab (Figure 2C), there are also annotation properties such as references, comments and curators notes. For more specific categories of information, we created specialized forms which that incorporated the basic information along with additional properties specific to (1) neurons, (2) brain regions, and (3) resources (See supplemental Table S3). The generic form is created anytime a user creates a new category page, using any of the methods available from the Semantic MediaWiki. The specialized forms are invoked when users provide an appropriate super-category within the generic form, e.g., entering “Neuron” under the super-category page will invoke the detailed neuron form. Users may also invoke these special forms by utilizing special category boxes available from the home page (Figure 1).

In addition to extensions that were already available in the public domain, we constructed some custom extensions to enable automatic generation of identifiers for each category/entry, a version number for each page, updated each time an edit is made, and a tool to allow categories to be uploaded via a comma separated value (CSV) text file. A full list of the extensions used in NeuroLex can be found online (http://neurolex.org/wiki/Special:Version). In order to allow search engines such as Google to index our page content, we customized the information in the “description” meta-tag in the header of each page to display text that was specific to each page.

We have set NeuroLex to export a complete RDF graph of the entire contents of the wiki and upload them to an installation of the Jena triple store (http://neurolex.org/wiki/NeuroLex_SPARQL_endpoint) once every hour. This enables users to access the semantic backend programmatically via the SPARQL language.

As detailed in the results section, while many questions can be asked using Semantic MediaWiki's native query language, we initially found ourselves limited by an inability to pose queries that could handle an arbitrary number of transitive operations in a single query (http://j.mp/A8V3YJ). To address this, we imported the RDF graph from NeuroLex into an instance of the OWL-IM semantic repository (http://www.ontotext.com/owlim) installed on Amazon EC2 (http://neurolex.org/wiki/Reasoning_With_SPARQL_1.1). We then utilized SPARQL 1.1 (http://www.w3.org/TR/sparql11-query/#propertypaths) queries to explore the knowledge base and answer specific questions regarding connectivity across brain regions.

## Results

NeuroLex.org has made progress toward establishing and enabling the creation of a comprehensive corpus of machine-processable, multi-scale neuroscience knowledge that is editable collaboratively online and is discoverable on the Internet by search engine queries. The following results reflect the state of the NeuroLex as of March, 2013.

NeuroLex currently hosts ~25,000 active distinct entries including ~700 neurons (Table 2) and ~900 brain regions, a threefold increase of concepts entries since its original launch (http://j.mp/xJnXej). The neurons span vertebrate and invertebrate species including drosophila, honeybee, mouse, rat, macaque, and human, while the brain regions are mainly specified in vertebrate species. Table 1 shows a high-level overview of the contents of NeuroLex.org, broken down by the high-level categories spanning scales and domains relevant to neuroscience.

**Table 1:**
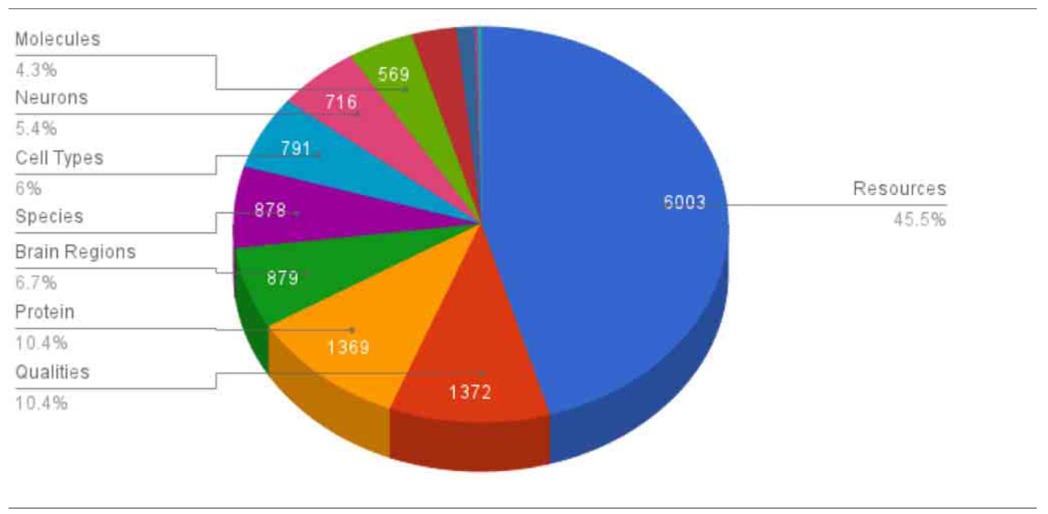
**Overview of key contents in NeuroLex**.

Pie sections not labeled after molecules include Diseases (385), Subcellular parts (151), Nervous system functions (35) and Behavioral activity (31). The latest content overview can be found online (http://g.ua/WCPs).

**Table 2:** **Nervous system cells in the NeuroLex**.

**Category**	**Definition**	**No. of terms**
Neurons	The basic cellular units of nervous tissue. Neurons are polarized cells with defined regions consisting of the cell body, an axon, and dendrites, although some types of neurons lack axons or dendrites.[…]	716
Glial cells	A non-neuronal cell of the nervous system. They not only provide physical support, but also respond to injury, regulate the ionic and chemical composition of the extracellular milieu. Guide neuronal migration during development, and exchange metabolites with neurons.	41
GABAergic neurons	A neuron that uses GABA as a neurotransmitter.	68
Glutamatergic neurons	A neuron that uses glutamate as a neurotransmitter.	41
Cholinergic neurons	A neuron that uses Acetylcholine as a neurotransmitter.	17
Dopaminergic neurons	A neuron that uses dopamine as a neurotransmitter.	6

An example page within the NeuroLex is shown in Figure 2. In this example, structured knowledge about the cerebellum is assembled and displayed on a web page that can be bookmarked or shared via the unique URL. With the custom extensions built for NeuroLex, either the identifier or the textual category name will resolve to the same page. As NeuroLex is built on top of MediaWiki software, an edit button is present that allows any user to modify the contents on this page (B). The default edit mode is via the form but users can chose to edit via the native wiki editor (“Edit source”).

The unique features of the Semantic MediaWiki platform are illustrated in Figure 2. On a standard Wikipedia page, all the knowledge on a page must be manually entered within the single text box provided. Because NeuroLex leverages a semantic backend to structure knowledge, its pages can dynamically call information from other pages when it is relevant. Boxes corresponding to (D), (E), and (F) demonstrate the ability of the NeuroLex.org infrastructure to assemble knowledge related to the cerebellum automatically. For example, in (D), all neurons reporting their somas to be within the cerebellum, or within any other brain region that is defined as *Is part of* the cerebellum (as shown in E), is listed here. The list is assembled via Semantic MediaWiki's inline query functionality, which allows structured content from other pages to be organized and reported. Additionally, any cell that reported that its axon passes through the cerebellum or its parts is listed separately. This information is not entered on this page, but is created from edits made to other pages, e.g., if a user enters a soma location for a neuron that is a part of cerebellum, the neuron automatically shows up on this page under the “Neurons found in cerebellum” section.

The ability to structure knowledge within the Semantic MediaWiki platform also allows classes to be rendered using tables, trees, and lists that combine the asserted content of the class with queried content derived from other classes. For example, (E) in Figure 2 displays a dynamic tree that lists the brain region classes that have been asserted as *Is part of* the Cerebellum. Lastly, (F) shows tracts that have been defined as afferent to or efferent from the cerebellum.

### Editing the neurolex

**Figure 3:**
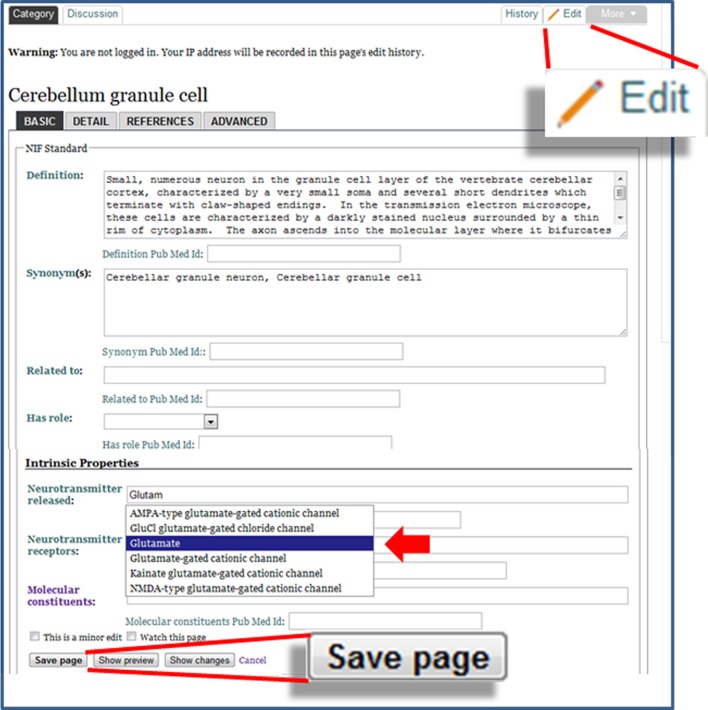
**The edit form for the Cerebellum granule cell page**. The form is invoked by clicking the Edit button, shown in the enlargement in the upper right. Text boxes enable the user to make edits to the fields of information on the page. Fields whose values link to other category pages have an autocomplete feature, which may be further refined through defining a domain restriction. In the example shown here, the “Neurotransmitter receptors” field selects from subclasses of “Molecule” (solid arrowhead). The save button at the bottom of the page makes the edits immediately visible in NeuroLex.

To enable users to make modifications to knowledge that has been structured within NeuroLex.org, we implemented a form-based edit system as opposed to the standard free text and markup system used by many wiki sites, including Wikipedia. Figure 3 shows an example of editing the page for a cerebellum granule cell, utilizing the specialized neuron form (http://j.mp/xzUwKR). In addition to being simpler to learn, NeuroLex facilitates a more interconnected knowledge base via two key features: red links and autocomplete. When a page is edited and saved, values associated with the concept on that page may appear in one of three colors, black, blue or red. Black text indicates a value that doesn't link; this is usually reserved for values or qualities that are provided in free text. Blue text indicates an active link to another concept entry also stored within NeuroLex; clicking this link will take the user to this entry, thus enabling discovery of related entries. Red text, referred to as red links, show a value that has the potential to link to another concept entry within NeuroLex, but a category page for the conceptentry does not currently exist. At this time, however, NeuroLex does not autocomplete on synonyms, so a red link may also indicate that the term used is not the official category name. A red link can occur as a result of a misspelling the concept's entry's name as supplied by the user, or the concept entry could be missing absent from in NeuroLex altogether. In the first case, the red text alerts the user that they may have made an error, and may need to edit the value further to cause it to turn blue. In the second case, the user may click on the red text to arrive at a blank page where they can define the missing feature or concept entry. While the red link doesn't tell these cases apart, in both cases this mechanism provides feedback to a user about how they can improve the knowledge base—rather than have their bit of knowledge sit alone and by itself, they can turn a red link into blue text and add greater organization to the framework as a whole.

Any user may edit a NeuroLex page, with or without an account, though the abilities to delete and to move pages are restricted to users with accounts. This permissive approach to edits is balanced by a built-in history and change tracking mechanism that makes edits transparent to curators, who can easily undo changes that negatively impact the quality of the knowledge base. The main curators of the NeuroLex site currently are part of the NIF project, all of whom have administrative privileges. Change history is available both at the level of individual pages (http://j.mp/zC8Aci) or at the level of the entire site (http://neurolex.org/wiki/Special:RecentChanges).

NeuroLex.org has been online since December 2008. In an analysis performed on March, 2012, it had received 280,182 absolute unique visits and 733,040 page views from 191 countries and territories. Currently NeuroLex is receiving ~600 hits per weekday. One hundred and three users have made edits, 31 of them have been active in the process of editing in the last 2 months. NeuroLex has recorded 204,667 edits providing a ratio of visits to edits of ~1.4:1 and a ratio of edits to content of 11:1 (Wikipedia has 14:1) (Spinellis and Louridas, 2008).

**Figure 4:**
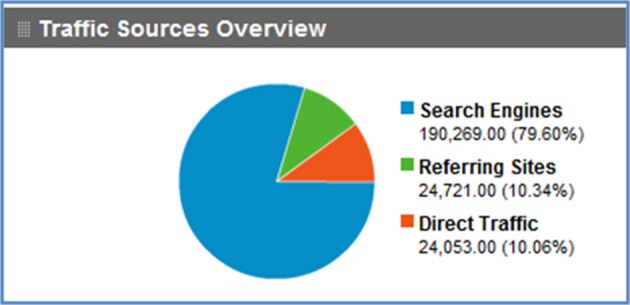
**Traffic sources to NeuroLex.org since December 2008**. Direct traffic refers to a user typing “neurolex.org” into the browser or following a personal bookmark. Referring sites are visits where a user started at another site and clicked a link to arrive at NeuroLex.org. Search engines refer to any user that came to NeuroLex.org from a web search. Google searches made up 95% of the search engine traffic.

**Figure 5:**
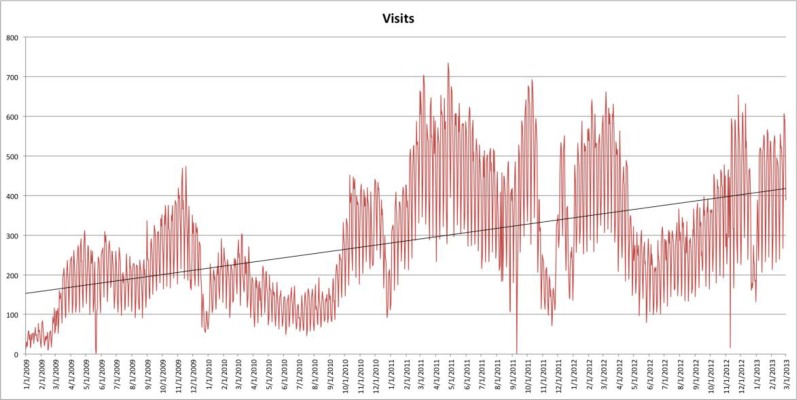
**A graph of visits to NeuroLex.org over time since December 2008**. Hits in 2010 were depressed by modifications in the presentation of metadata for search engines. This was corrected at the end of 2010, which led to increased traffic seen in 2011. Traffic dipped again briefly at the end of 2011 because of a site configuration error that was corrected early 2012. A trend line is added to show average traffic changes over time.

As shown in the usage graph in Figure 4, Google search has had a strong impact on traffic flow to NeuroLex.org, bringing the majority of traffic to the site. Modifications to the way that NeuroLex reports the contents of each page to Google resulted both in a reduction of average traffic at the end of 2009 and a sharp increase of average traffic at the end of 2010 (Figure 5).

In an analysis performed in January of 2012, some 979 distinct terms when searched in Google returned a NeuroLex page in the first 10 results. These include terms such as “dorsal root ganglion,” “telencephalon,” “nervous system function,” “cholinergic neurons,” “mni atlas,” “mitral cells,” “primary olfactory cortex,” “lateral septum,” “movement quality,” and “oddball paradigm.” However, those top 10 hits make up only 3.8% of the 190,000 visits from Google. In fact, 96.2% of the visits come from searches for 87,047 other keywords. Because of this, NeuroLex.org searches from Google have a “long tail” (Anderson, 2004) quality to them where the value of the content is the comprehensive catalog of items that are held rather than the popularity of a few major terms, a feature that has been observed in other biological wiki efforts (Huss et al., 2008).

### Usage and adoption

As described in Imam et al. (2012), in order to enable broad community contribution to the NIF Standard Ontology (Bug et al., 2008), the Neuroscience Information Framework adopted NeuroLex.org and made it available as an easy entry point for the community (Grethe, 2009; Figure 1). The NeuroLex has become the community facing front-end to the NIF Standard Ontology as well as the platform on its comprehensive registry of neuroscience resources is hosted. As content is modified and updated on NeuroLex.org, an ontology engineer tracks the latest changes, reformulates them into a stricter formalism if necessary, and updates the OWL representation of the NIF Standard Ontology. The NIF Standard Ontology then serves as the back-end for concept-based search that is deployed to the NIF search engine (Imam et al., 2012). The Semantic Wiki platform provides the ability to embed additional tools and widgets to link NeuroLex content to other resources. For example, the NIF Navigator, which presents a result of a NIF query organized by data type and level of nervous system has been embedded on each page, so that users immediately link to additional data sources about the concepts entries represented in within NeuroLex.

The NeuroLex is available for use by the community and provides a convenient platform for groups working on vocabularies to expose their content. For example, French et al. (2009) developed an algorithm for extracting brain regions from the literature. They utilized NeuroLex, NeuroNames and The Brain Architecture Management System [BAMS; Bota et al. (2005)] to develop the training set and extracted additional terms not present in these resources. Using the bulk spreadsheet upload, they contributed them back to the NeuroLex under the super-category “Brain regions extracted through automated text mining.” As the NeuroLex is curated, these terms are naturally viewed by human curators who further incorporate them into the NeuroLex as needed.

NeuroLex.org has also served as a test bed for the efforts of the Program of Ontologies of Neural Systems (PONS; Martone et al., 2010), an activity of the International Neuroinformatics Coordinating Facility (INCF)^2^ The original intent of this group was to explore ways to create an on-line reference work where structured information on brain regions and neurons could be immediately accessible and editable by the community at large. Unlike Wikipedia, which requires structures to be notable before they are included, common to any encyclopedia^3^, the PONS program wanted a place where machine processable definitions of any brain structure, spanning the range from macromolecules to brain regions, could be built by non-expert ontologists, in a way that facilitated their use within information systems. To support these efforts, two customized forms with a defined set of properties were created: PONS Brain Region and Petilla Neuron [based on the proposal in Ascoli et al. (2008)]. Details of these activities will be presented in separate reports (e.g, Hamilton et al., 2012). In the following sections, however, we describe some of the native and custom features of NeuroLex in support of these types of use cases.

### Relations

**Figure 6:**
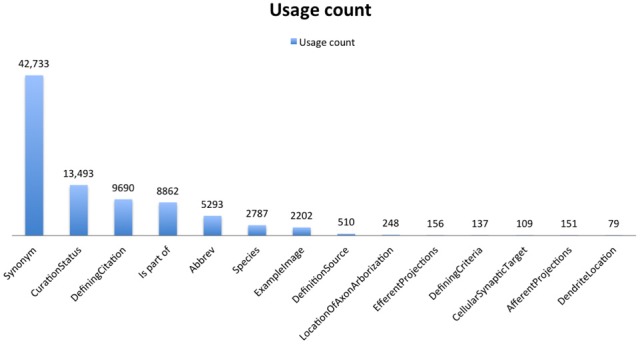
**Overview of key relations**. Derived from data that appears on the web at http://neurolex.org/wiki/Special:Properties.

**Figure 7:**
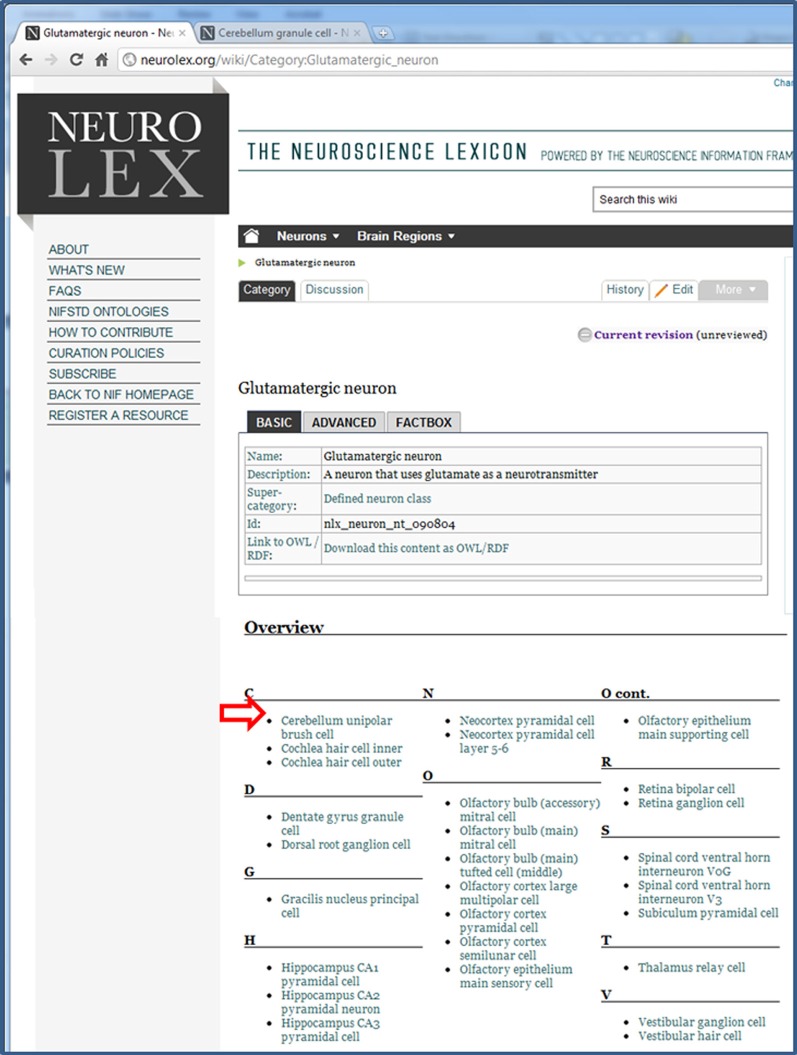
**The page for all Glutamatergic neurons**. This page is a defined neuron class, which means that it is a collection of neurons that come together as a result of a shared property, the presence of glutamate as its neurotransmitter. The neurons within NeuroLex for which this is true are listed in the Overview section, which starts with the open arrowhead.

**Figure 8:**
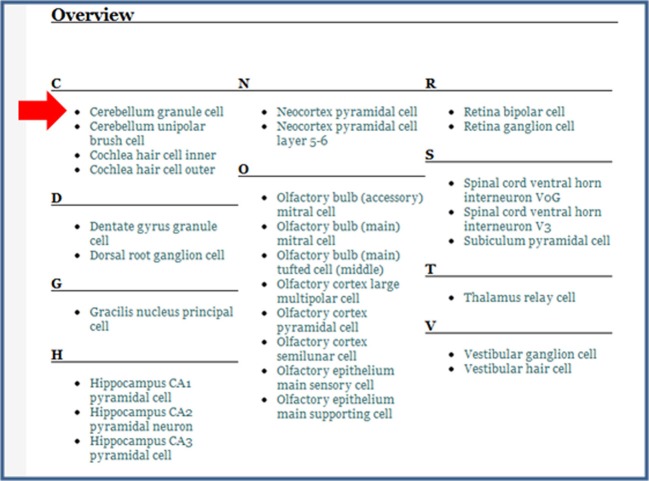
**The modified overview section of the Glutamatergic neuron page**. After having entered Glutamate as the Neurotransmitter released in the Cerebellum granule cell page (Figure 3), this neuron now appears in the list when it did not before (compare with open arrowhead in Figure 7 above).

Each of these forms contains a specified set of properties, that can be implemented either as a page or data level property, according to the Semantic MediaWiki convention. A page property links together two category pages; a data type property allows quantitative information or string values to be entered, but does not automatically link category pages. Fields can contain multiple values, separated by commas, which create separate RDF triples for each value provided. Thus, as shown in Figure 6, the number of synonyms far exceeds the number of category pages (currently totaling more than 25,000), as each concept entry can have multiple synonyms. The properties chosen for each category (Supplemental Table S3) were not meant to be exhaustive within the domain, but they do represent a consensus view from multiple experts on the key attributes. These attributes form the basis of automated embedding information within related pages and generating additional classifications within the NeuroLex. As shown in Figure 2, entities related to the cerebellum such as neurons and axon types are automatically inserted within the page as they are added in other pages using the MediaWiki template mechanism. Similarly, additional classifications can be generated from the NeuroLex to place a particular concept entry in multiple categories through automated means. The NeuroLex tries to enforce the single asserted hierarchy constraint recommended by many in the ontology community (Rector, 2002). By single asserted hierarchy, we mean that each category is asserted to be a member of only a single super-category (parent class). However, following conventions from the ontology community, we provide for the ability to create defined classes, that is, classes whose memberships is determined by an execution of a rule. For example, Figure 7 illustrates a page in NeuroLex that only lists neurons that are defined to have glutamate as their main neurotransmitter. Rather than editing this page directly in order to maintain this list, the page is updated automatically by the system when a user edits the page of an individual neuron and indicates that its neurotransmitter is glutamate (Figures 3, 8, filled arrowhead). To create this page, the following text specifying that subclasses of the neuron category that satisfy the neurotransmitter constraint should be shown on this page:

{{#ask: [[Category:Neuron]]

[[Neurotransmitter::Category:Glutamate]]

|format=category

}}

This and similar text have been copied and pasted by non-technical users to create custom lists of other types of specialized categories of items, e.g., hippocampal neurons, parvalbumin neurons, rodent brain parts, human brain parts. The results of this automatically generated list also becomes visible to search engines, meaning that a list that is useful to an individual may also become content that is reused by others. For example, at time of writing, the custom list on NeuroLex for “cholinergic neurons” appears on the first page of results in Google.

### Cerebellum reasoning example

The original intent of the NeuroLex was to provide an interface to enhance the concept-based search functionality of the NIF system for query over distributed data sources. Thus, information supplied by contributers to the NeuroLex has been incorporated within NIFSTD and exposed through the NIF. Thus, when a user queries NIF for GABAergic neuron, it retrieves data on all neurons that have been classified within NeuroLex as GABAergic. However, as the NeuroLex has grown, it has become a significant knowledge base of basic information about the nervous system in and of itself. In order to validate the ability of NeuroLex to structure information in a form that enabled the answering of significant questions about the nervous system, we explored the capacity of the knowledge base to answer the question: “What are all the brain regions that send projections into the cerebellum or any of its parts via mossy fibers?” As detailed in the methods section, this query could not be handled by the innate query functionality of the current wiki, but required importing the NeuroLex knowledge graph to a triple store that supported SPARQL 1.1. To address this question, we included statements derived from a textbook on cerebellar anatomy (Altman and Bayer, 1997) in order to give the system a means of understanding (1) the parts of the cerebellum, (2) what it means to send projections, and (3) what mossy fibers are. In this case, mossy fibers are implied by any axons that enter the cerebellum, or any projection whose destination is the cerebellum, other than explicitly defined climbing fibers.

The query issued is shown in Table 3, while the results are shown in Table 4. Here we have asked for the names of all brain regions that send axons into the cerebellum. The query takes advantage of the “AfferentProjections” and “EfferentProjections” properties, the “SomaLocation” and “LocationOfAxonArborization” property, as well as more standard relationships such as “subClassOf” and “isPartOf.” We specified “the cerebellum” as all the parts of the cerebellum that can be reached via the “isPartOf” relationship, which numbered 65 different areas.

**Table 3:**
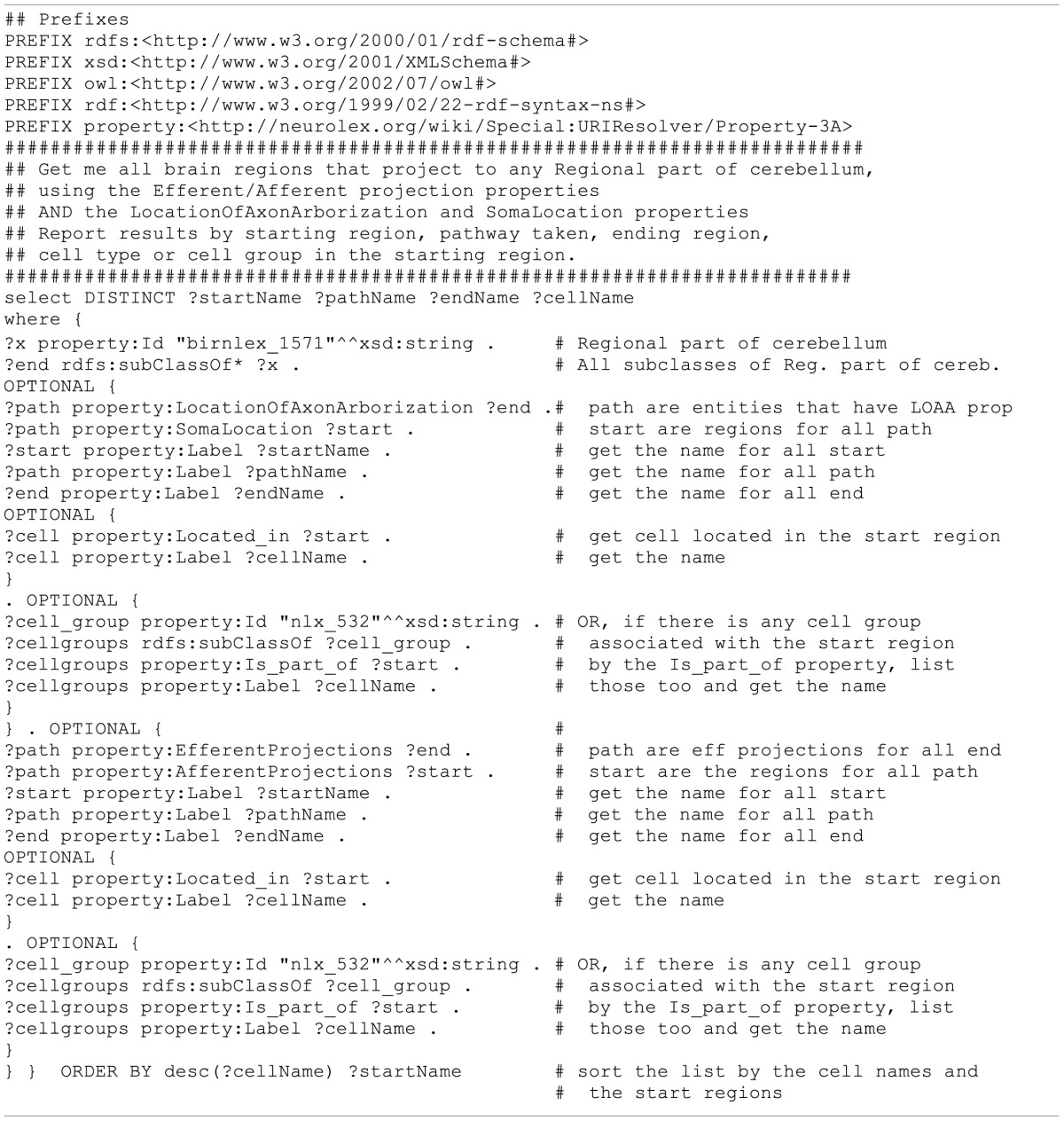
**SPARQL 1.1 query to return the brain regions that project into the cerebellum**.

**Table 4:** **Results from performing the query in Table 3 to answer the question what brain regions project to some part of the cerebellum**.

**Location of cell soma**	**Pathway taken**	**Location of axon arborization**	**Cell type**
Vestibular ganglion	Cerebellar Primary Vestibular Afferents	Hemispheric Lobule IX	Vestibular ganglion cell
Vestibular ganglion	Cerebellar Primary Vestibular Afferents	Hemispheric Lobule VIII	Vestibular ganglion cell
Vestibular ganglion	Cerebellar Primary Vestibular Afferents	Vermic Lobule I	Vestibular ganglion cell
Vestibular ganglion	Cerebellar Primary Vestibular Afferents	Vermic Lobule IX	Vestibular ganglion cell
Vestibular ganglion	Cerebellar Primary Vestibular Afferents	Vermic Lobule X	Vestibular ganglion cell
Motor nucleus of trigeminal nerve	Trigeminal Mossy Fibers	Hemispheric Lobule VI	Trigeminal nucleus motor neuron
Motor nucleus of trigeminal nerve	Trigeminal Mossy Fibers	Hemispheric Lobule VIIBi	Trigeminal nucleus motor neuron
Motor nucleus of trigeminal nerve	Trigeminal Mossy Fibers	Hemispheric Lobule VIII	Trigeminal nucleus motor neuron
Motor nucleus of trigeminal nerve	Trigeminal Mossy Fibers	Vermic Lobule IX	Trigeminal nucleus motor neuron
Pontine reticular formation	Cerebellar Afferents From The Pontine Reticulotegmental Nucleus	Anterior lobe of the cerebellum	Serotonergic cell group B8
Pontine reticular formation	Cerebellar Afferents From The Pontine Reticulotegmental Nucleus	Hemispheric Lobule VII	Serotonergic cell group B8
Pontine reticular formation	Cerebellar Afferents From The Pontine Reticulotegmental Nucleus	Hemispheric Lobule VIII	Serotonergic cell group B8
Pontine reticular formation	Cerebellar Afferents From The Pontine Reticulotegmental Nucleus	Paravermic Lobule VII	Serotonergic cell group B8
Pontine reticular formation	Cerebellar Afferents From The Pontine Reticulotegmental Nucleus	Paravermic Lobule VIII	Serotonergic cell group B8
Pontine reticular formation	Cerebellar Afferents From The Pontine Reticulotegmental Nucleus	Vermic Lobule VII	Serotonergic cell group B8
Pontine reticular formation	Cerebellar Afferents From The Pontine Reticulotegmental Nucleus	Vermic Lobule VIII	Serotonergic cell group B8
Cervical spinal cord	Central cervical spinocerebellar tract	Vermic Lobule II	N/A
Cervical spinal cord	Central cervical spinocerebellar tract	Vermic Lobule III	N/A
Cervical spinal cord	Central cervical spinocerebellar tract	Vermic Lobule IV	N/A
Cervical spinal cord	Central cervical spinocerebellar tract	Vermic Lobule V	N/A
Cervical spinal cord	Central cervical spinocerebellar tract	Vermic Lobule VI	N/A
Cuneate nucleus	Posterior spinocerebellar tract	Paravermic Lobule IV	N/A
Cuneate nucleus	Posterior spinocerebellar tract	Paravermic Lobule V	N/A
Cuneate nucleus	Posterior spinocerebellar tract	Paravermic Lobule VI	N/A
Cuneate nucleus	Posterior spinocerebellar tract	Vermic Lobule IV	N/A
Cuneate nucleus	Posterior spinocerebellar tract	Vermic Lobule V	N/A
Cuneate nucleus	Posterior spinocerebellar tract	Vermic Lobule VI	N/A
Lateral reticular nucleus	Cerebellar Afferents From The Lateral Reticular Nucleus	Anterior lobe of the cerebellum	N/A
Lateral reticular nucleus	Cerebellar Afferents From The Lateral Reticular Nucleus	Hemispheric Lobule VIIBii	N/A
Nucleus prepositus	Cerebellar Afferents From The Prepositus Nuclear Complex	Cerebellar hemisphere	N/A
Nucleus prepositus	Cerebellar Afferents From The Prepositus Nuclear Complex	Hemispheric Lobule IX	N/A
Pontine nuclear complex	Cerebellar Pontocerebellar Projection	Hemispheric Lobule VI	N/A
Pontine nuclear complex	Cerebellar Pontocerebellar Projection	Hemispheric Lobule VIIBi	N/A
Pontine nuclear complex	Cerebellar Pontocerebellar Projection	Hemispheric Lobule VIII	N/A
Vestibular nuclear complex	Cerebellar Secondary Vestibular Afferents	Hemispheric Lobule IX	N/A
Vestibular nuclear complex	Cerebellar Secondary Vestibular Afferents	Hemispheric Lobule VIII	N/A
Vestibular nuclear complex	Cerebellar Secondary Vestibular Afferents	Vermic Lobule I	N/A
Vestibular nuclear complex	Cerebellar Secondary Vestibular Afferents	Vermic Lobule IX	N/A
Vestibular nuclear complex	Cerebellar Secondary Vestibular Afferents	Vermic Lobule X	N/A
Vestibular nuclear complex	Cerebellar Secondary Vestibular Afferents	Vermic Lobule I	N/A
Vestibular nuclear complex	Cerebellar Secondary Vestibular Afferents	Vermic Lobule IX	N/A
Vestibular nuclear complex	Cerebellar Secondary Vestibular Afferents	Vermic Lobule X	N/A

This query has also taken advantage of the “LocatedIn” relationship that relates the cell bodies of neurons to brain regions. Using this property we are able to identify cell types that may be responsible for the axons projecting between regions. For several regions, there are no putative neurons known to the system yet—this highlights a gap in the knowledge base that can be addressed in the future.

Finally, the query also searches across any “cell groups” (http://neurolex.org/wiki/nlx_532) associated with a brain region via the “isPartOf” relationship, to further reveal any cell types that may be responsible for axons leaving that region. This has returned to us a hypothesis that Serotonergic cell group B8 may be involved in the projections from the Pontine reticular formation to several lobes and lobules of the cerebellum.

As a comparison, we also performed similar queries against other on-line databases that contain information about connectivity: The Brain Architecture Management System [BAMS; Bota et al. (2005)] BAMS and the CoCoMac database (Kötter, 2004). The equivalent query to find the incoming projections to the cerebellum in the BAMS database reveals a list of 3 brain regions that project into the lateral lobes of the cerebellar cortex and 27 brain regions that project into the flocculus (http://j.mp/zKBZOB). BAMS reports 5 regions that project into the cerebellum as a whole (http://j.mp/xoWZMG). NeuroLex can report the pathway or cell type that is responsible for the projection. A similar report can be generated from the web interface of BAMS^4^. The CoCoMac database does not store connections to the cerebellum, so the equivalent query could not be run.

The system used to create these queries, with the version of the data used to call for them, is available for inspection online (http://neurolex.org/wiki/Advanced_SPARQL_Queries).

## Discussion

The production of a well structured and comprehensive “parts list” or knowledge base that is machine processable would be a key asset to the field of neuroscience as it would drive hypothesis generation across subdisciplines. Significant contributions in collating neuroscience knowledge into information systems have been made over the last few decades, e.g., BAMS, BrainInfo, CoCoMac, SUMSdb. However, these resources are maintained by individual curators, who ensure the consistency and quality of the knowledge base. The advent of widespread usage of the Internet as the primary means of conducting research online has made it possible to explore entirely new means of building both broad, deep, and organized corpuses of knowledge in a distributed collaborative manner (Neumann and Prusak, 2007; Huss et al., 2008; Spinellis and Louridas, 2008). The great magnitude of increase in potential interaction of large numbers of individuals over the Internet provides hope in solving the even greater challenge of building a detailed corpus of knowledge about the nervous system.

In order to tap into this potential, the exchange of knowledge in the biological sciences in the age of the Internet will increasingly demand tools that allow the organization, presentation, and dissemination of the complex relationships of living systems through interfaces that are easy to update and easy to use. Maintaining a careful balance between complexity and simplicity is a multi-disciplinary challenge. Addressing this challenge requires as much attention to the interests of biological scientists who do not have deep experience with information systems as it does to the interests of logicians and ontological experts, who have experience structuring knowledge in ways to allow automated query and reasoning. These interests, frequently in competition, make up two sides of a coin. If biological scientists cannot easily get knowledge out of an information system, they cannot benefit from it. If biological scientists cannot easily put knowledge into an information system, the system will be uninteresting for lack of content. At the same time, if ontological experts cannot structure queries and reason over domain knowledge, an information system will not be able to return interesting results or reveal non-obvious knowledge connections and will also suffer from disuse.

With NeuroLex.org, we have demonstrated success in building a community platform where neuroscientists, ontology engineers and knowledge managers can structure knowledge in a wiki form in an online repository that is accessible to everybody. We have also demonstrated success in producing an online information artifact that is useful for the discovery and organization of neuroanatomical facts. We invested effort into creating properties that allow knowledge to be appropriately interlinked for the purpose of creating a machine queryable semantic graph-based knowledge base that can connect facts at the micro-scale in neuroscience, to facts at a macro-scale. The system enables any user to look at different relationships between the entities through a built-in reasoning system. The knowledge base is still a work in progress—more facts are needed to fill in the significant number of gaps. However, as we have shown in the example of reasoning over facts of the cerebellum, the system is enabling us to ask much more powerful questions about facts that have been recorded in the literature.

The contribution model was designed to reduce the barrier to entry to allow and encourage a greater number of people to contribute. Although no wiki is entirely intuitive, we have seen from the usage of individuals we have worked with that the learning curve is significantly lower than current ontology editing tools and most customized database entry systems. Of equal importance, we have seen users with a minimal knowledge of ontology languages or programming skills report that the wiki platform allows them to create custom knowledge reports, something that typically requires an ontology expert or database programmer. Additionally, the NeuroLex exposes structured knowledge about neuroscience to the world via search engines, which unlocks the potential for many others to find and learn from the knowledge base we have created.

We have observed that neuroscientists increasingly use Google as a primary source of research. The traffic patterns NeuroLex has received from Google shows there is a broad worldwide demand for information about a variety of terms in neuroscience. With more than 4000 different online resources relevant to neuroscience on the Internet, according to the NIF Registry (http://neuinfo.org), it would be natural to assume that NeuroLex.org would rarely appear near the top of a Google search. However, much of the content of these resources is in dynamic databases, which are part of the “hidden web.” As we discovered with the traffic pattern dropping in 2010 and resurging in 2011 (Figure 5), optimizing a page for Google's search engine can make a big difference in the usage of an online resource. If Wikipedia is any guide, the more that scientists searching for scientific terms land on pages containing information that is useful to them, the more likely they are to begin contributing their own knowledge back to it (Spinellis and Louridas, 2008), thereby moving toward the conditions necessary to drive the vision of a community-vetted, collaboratively editing corpus of structured knowledge in the neurosciences to become a reality.

NeuroLex is actively used for enhancing the concept-based search capability of the NIF data federation and is also starting to be used by other groups. NeuroLex.org has been cited in a series of articles commentaries and reviews as a tool that can help address informatics challenges in neuroimaging (Nielsen, 2009b; Mejino et al., 2010; Turner et al., 2010), Autism (Young et al., 2009), event-related brain potentials (Frishkoff et al., 2009), and cognitive science (Derom et al., 2010; Miller et al., 2010; Yarkoni et al., 2010). The neuroinformatics community has cited it as a development encouraging integration between tools (French et al., 2009; Nielsen, 2009a; Ascoli, 2010; Hamilton and Ascoli, 2010; Katz et al., 2010; Akil et al., 2011). It has also been cited by the semantic web community as an example of a new development in distributed collaborative creation of biological ontologies (Cheung et al., 2009; Alquier et al., 2010).

### Contribution model, usability and interface

The issues surrounding the neuron input form made it clear that issues related to usability and interface, not commonly given first priority in scientific disciplines of informatics, are increasingly becoming crucial to enable online community interaction around scientific subjects. If we are serious about pursuing distributed collaboration, also called “crowdsourcing” (Howe, 2006) as a means of assembling complex knowledge bases in the biological sciences, then we must immediately view other successful crowdsourcing and social media ventures such as Wikipedia, Twitter and Facebook as models to follow. The organizations behind these sites make significant investments in interface and usability in order to produce easy-to-use experiences for their target audience. This acknowledges the reality of the contribution model on the Internet where sites like NeuroLex must compete for attention and for the dedication of its targeted users. These resources are difficult to get with clunky, cluttered, unintuitive interfaces that put a large cognitive burden or have a significant learning curve to use.

The NeuroLex, though having a relatively small number of active users who make edits, has an active user for every 1400 pages. To reach the ratio enjoyed by Wikipedia, which has an active user for every 210 pages would require adding an additional 68 active users, or in other words, persuading 1 out of every 300 unique visitors to the site to make an edit over the course of 2 months (the time window of user activity for them to be considered “active”).

The current NeuroLex interface has many features for encouraging community interaction (see Figures 1, 2). For example, the ease of bookmarking, linking to, and sharing content via unique resource locator (URL), unique resource identifier (URI) or unique id lowers the barriers to individuals discovering the site and returning to it. These unique identifiers are also important means for referencing these same features and concepts within other information systems, thereby facilitating interlinking and integration of knowledge.

The advantage of wiki platforms is that the edit features provide immediate gratification to users when making a contribution because they can see their edits are immediately visible globally. This approach is directly opposite to the tightly controlled nature of edits made to other ontologies as proposed by the OBO Foundry, another prominent group involved in structuring knowledge for the biosciences (Smith et al., 2007). For the OBO Foundry, only a small group of editors is allowed to make any changes, and a request system is used to make updates. OBO Foundry exposes their ontologies via the BioPortal (Noy et al., 2009), which has a mechanism for commenting or requesting modifications, rather than making edits directly to the underlying contents. This approach has been taken to ensure rigorous consistency of the knowledge-base with a gatekeeper model limiting the number of individuals allowed to make changes. In contrast, the NeuroLex wiki-based approach is to allow anyone to edit, and to deal with consistency after edits have occurred with a “Recent changes” list that makes it transparent exactly what edits have occurred when and by whom (see the link in (J) on Figure 2).

Of the ~30 ontologies managed by the OBO Foundry at their SourceForge repository, the combined number of requests on all trackers for all ontologies at time of writing was 3402, which makes for 55 NeuroLex edits (over 2 years on line) to every OBO Foundry request for modification (over 6 years of operation at SourceForge.com). Put another way, the OBO Foundry ontologies have been requested to be edited on average 1.5 times a day while the NeuroLex ontology has been edited on average 258 times a day. While more edits does not immediately suggest a higher quality of content, a difference of two orders of magnitude suggests a difference in the scalability of the contribution model.

### Knowledge base quality

As we have considered NeuroLex both as a platform for helping a community and as an information artifact, the question of knowledge base quality has arisen. The open nature of NeuroLex raises potential concerns about the completeness and accuracy of its content. In addition to building the NeuroLex on the software stack of Wikipedia, we have also built the contribution model on the foundation that underlies the success of Wikipedia in ensuring the breadth and accuracy of its articles (Giles, 2005). The idea is simple, the easier it is to edit and the more transparent those edits are to others, the more possible it is for the readership of the resource to also act as the reviewers and editors of content. However, since the concept entries are constantly changing and not rigorously peer-reviewed, they are not intended to be cited in journals as authoritative. Rather, wherever possible, we have included the ability to cite a publication elsewhere via a Digital Object Identifier (DOI) or a PubMed ID, or to otherwise indicate its original source from another resource, such as BrainInfo, BAMS, or published books. These citations are intended to enable and encourage the end user to find the publication from which the facts have been derived, confirm their veracity, and use these references as the authoritative, citable reference. Nonetheless, we believe that the integration of information within NeuroLex is its major strength, much in the way that the index of a book enables a quick lookup of concepts. The value of an index is derived from its ordering of concepts and the correctness of its pointers to pages and, via the NIF, to information sources that contain data relevant to the entity. Thus, the value of NeuroLex is derived from organizing the defining anatomical features and principal concepts of neuroscience into a data model and its accurate pointers to external references.

### Relations/properties

Understanding the importance of well-defined relationships is crucial to the mission of creating computer frameworks to grapple with the complexity of biological systems (Smith and Rosse, 2004; Smith et al., 2005). Indeed, these relationships are the glue that hold the knowledge base together—they are the edges that connect the vertices of the complex web of interactions that must exist between the biological entities playing out their roles within biological systems. For example, once you define a neuron as an entity of interest in a computer system, you are presented with the challenge of defining the set of relationships that this neuron should have to other entities—essentially what are the properties of a neuron? Despite more than 100 years of investigation, this is a challenge that is mostly unrecognized by the neuroscience community, and for which no consensus has yet been established by those in the biomedical ontology community (Migliore and Shepherd, 2005; Bota and Swanson, 2007; Ascoli et al., 2008; Hamilton et al., 2012).

The challenges in defining the proper relationships for a neuron to provide a complete description fall into multiple areas of concern: simplicity, exhaustive completeness, and computability. The concern for simplicity is that the set of relationships a neuron have should not be so large that no neuroscientist is able to contribute knowledge about a neuron because the number of things they need to fill out is daunting and overwhelming. Additionally, if the relations are too simplistic they may not fully describe the aspect of reality they intend to, much like describing a number as being “between one and a hundred” when the value 55 is really what is needed. On the other hand, exhaustive completeness is a concern for the utility of the framework—without all the details about a neuron, there will be unnecessary gaps that will prevent valuable insight to be gained. Related to this concern, but still separate, is the concern over computability. Here the concern is over the formality of the relations—if formal statements can be used, then more expressive questions can be asked via first order logic operations (Imam et al., 2012).

The need for simplicity is illustrated by our experiences in developing the Neuron form. Several considerations went into the development of the current list. An initial version adopted the more extensive recommended property list proposed by the Petilla convention for interneuron properties (Ascoli et al., 2008). While this provided a fairly complete overview of relevant properties, users reported difficulty making contributions to the NeuroLex with the number of properties was too high or when the values to be supplied were unclear. This highlighted the reality that even when defining a single neuron, different potential contributors to the knowledge base will disagree on the importance of certain properties, and the values they should have. In pursuit of a more parsimonious set of properties, the current list was produced to include more multiple choice options and fewer numerical values (e.g. Cell soma size, Firing rate). While the NeuroLex has taken the approach of setting the template for all users, a new effort to create a Neuron Registry has explored the possibility of allowing users to flexibly define and use properties as needed (Hamilton and Ascoli, 2010). While both approaches have their pros and cons, data on which approach is best is still limited.

### Organizing structured knowledge online

Wikipedia's approach to exposing structured knowledge has been through info-boxes that are implemented via MediaWiki templates. The DBPedia project (Hahn et al., 2010) has mined these info-boxes to package the knowledge within them into RDF. With NeuroLex, we have circumvented the need for this two step process, first to an info-box, and second to RDF, by building on top of Semantic MediaWiki. The use of Semantic Forms by NeuroLex eliminates the need for the user to learn wiki-text syntax, as Wikipedia editors must. Users are always presented with a form rather than a block of text when they click the edit button in NeuroLex, unless they specifically request otherwise.

In some cases, however, we found limitations of Semantic MediaWiki in providing functionality that we view as necessary for the effort to reconcile neuroscience knowledge into digital forms. In particular, we found a need to have better ways to work with synonyms because users need to be able to arrive at the page for a subject from multiple URL entry points—by default the Semantic MediaWiki platform doesn't automatically generate redirect pages that could facilitate this. Additionally we found a need to better support inheritance and reciprocal relations, functionality that would enable additional reasoning capabilities within the wiki without requiring the use of an external semantic processor. Finally, the need to be able to associate a citation to any arbitrary triple also stretches beyond the default capacity of Semantic MediaWiki, a feature that would assist in making all facts in the knowledge base point back to an authoritative reference.

On the whole, however, after considering other wiki platforms, we still chose Semantic MediaWiki over other similar platforms such as AceWiki (Kuhn, 2008), Confluence, or Wikipedia itself. AceWiki is a system that supports natural language and first order logic operations within a wiki context. Here we found that the community supporting this tool was too small and this created significant risk that there would be few community tools and support to utilize. Confluence is a popular open source wiki product built by Atlassian, however, no semantic extensions to it have been built as far as we know. Lastly, the approach of the GeneWiki (Good et al., 2011) has been to leverage Wikipedia itself. While we explored this option, we felt that the flexibility of building on our own servers and being able to make modifications to the platform without special permission from the Wikimedia foundation enabled us to prototype functionality much more rapidly than would have been possible on that platform.

### Cerebellum reasoning example

In order to demonstrate that the structuring of knowledge in the neurosciences could help to provide answers to questions that were not obvious, we exported the RDF graph of NeuroLex into a triple store environment that allowed us to pose a specific query (Table 3) and retrieve results that were pulled from the pages of the wiki (Table 4). We have exported this content outside of the Semantic MediaWiki framework in order to take advantage of advanced query operations and additional computational performance only available in a separate installation of a SPARQL 1.1 compliant triple store (OWLIM).

Three efforts to enable users to aggregate and investigate connectivity relationships are related to this investigation: CoCoMac, BAMS, and the ConnectomeWiki. These resources can be compared from the perspectives of access control, style of representation, and ability for external programmatic interface. Both the CoCoMac and the BAMS approaches use carefully curated and restricted access approaches to including new connectivity statements in their database. Neither of them allows users to contribute to the knowledge base without a vetting process. In contrast, the ConnectomeWiki project took a similar approach to NeuroLex in terms of access control. Both allow anyone with a user account, available for free without special permission, to make edits to their database of connectivity statement. In addition, the NeuroLex allows anonymous users to make edits as well. One different between NeuroLex and the ConnectomeWiki is that the presence of separate pages that capture information about connections in the ConnectomeWiki allowing connections to be individually named and referenced. This is an extremely useful addition that has not yet been incorporated into NeuroLex, although as the example demonstrates this is not a pre-requisite for reasoning about connectivity.

CoCoMac and BAMS have both built their knowledge bases as database schema models, which limit the ability to create open and flexible linked data models (Ruttenberg et al., 2009). NeuroLex and the ConnectomeWiki use a semantic data model. A database schema model is a series of tables with rows and columns, while a semantic data model is a directed graph. Queries on directed graphs can be written using the logic of the properties that make up the edges of the graph and the question in mind, rather than the logic of a database schema via SQL. Using the logic of the semantic data model is useful for building a query up through an interactive process of query and exploration by the user. Graph queries also make some operations more intuitive to work with, such as the ability to recursively search through all subclasses of “regional part of cerebellum,” an operation that is much more verbose to express using SQL. Using the same formalisms, we have also been able to answer questions about how complete the knowledge base is (Supplemental Table S1) for neurons and to reclassify neurons by their neurotransmitter.

This example demonstrates the underlying motivation for the effort we have put into NeuroLex. The purpose of aggregating knowledge in a structured manner is to make it possible to ask questions that shed new light on facts we have already acquired. Because of the inherent complexity of the nervous system, it is difficult for any single investigator to keep the totality of its understanding in mind. This has necessarily led to specialization in the neurosciences and a balkanization of knowledge, which makes questions that cross between specialties hard to answer. With the NeuroLex, we have provided a system that can allow many different investigators to be aggregate their specialized knowledge into a form where questions that cross their specialties can be answered. While each individual fact may already be known, in the aggregate, and with advanced query mechanisms, it is possible to put into the hands of curious investigators the tools to ask and answer precise questions they are looking for, rapidly and without the work of aggregating the knowledge themselves. More importantly, all concepts entries can be linked to the literature or data sources that can be used for further investigation.

This kind of built-in organization based on adding knowledge necessary in the biosciences because of the complexity of the entities we are trying to describe. Due to the facility to interlink knowledge into a graph, this kind of system can act as a counterweight to complexity—every piece of knowledge does not stand on its own, rather it creates a more parsimonious explanation. The features we have described here appear to us to approach the qualities of the “magic index card” system described in the introduction.

### Conflict of interest statement

The authors declare that the research was conducted in the absence of any commercial or financial relationships that could be construed as a potential conflict of interest.

## Footnotes

^1^A term from computer science referring to the explicit management of data type within the text of the code, as opposed to allowing data type to be implicit and managed by the compiler. Declaring a variable to be a String in your code is evidence of a strongly-typed language. Languages that do not require the declaration of a variable as a string are referred to as “weakly-typed”.

^2^See: http://www.incf.org/core/programs/pons for more.

^3^ See: http://en.wikipedia.org/wiki/Wikipedia:Notability for more.

^4^http://brancusi.usc.edu/bkms/brain/fib-compoz.php?aidi=145
