# Supplementary material for: NeuroLex.org: an online framework for neuroscience knowledge
Source: Front Neuroinform. 2013 Aug 30;7:18. doi: 10.3389/fninf.2013.00018 (PMC3757470; doi:10.3389/fninf.2013.00018)
Supplement: Supplementary file 2 [file DataSheet2.PDF]

| | URL | Citation | Coverage | Wiki-based | Anyone can edit? | Staff curator? | Ontology backend | Can get contents in OWL/RDF | Can hyperlink to individual entries? | Different neuron parts associated with different brain regions? |
|---|---|---|---|---|---|---|---|---|---|---|
| NeuroLex | http://neurolex.org | | Neurons, other cell types, brain regions, connections, proteins & molecules, species, cognitive processes, qualities, subcellular components, nervous system functions, diseases, information resources | Yes | Yes | Yes | Yes | Yes | Yes | Yes |
| Neuron Registry | http://incfnrci. appspot.com/ | Hamilton and Ascoli, 2010 | Neurons | No | No | Yes | No | No | No | Yes |
| BAMS | http://brancusi.usc. edu/ | Bota et al., 2005 | Neurons, Brain regions, connections | No | No | Yes | Some | No | Yes | Yes |
| BioPortal | http://bioportal. bioontology.org/ | Noy et al., 2009 | | No | Some | Yes | Yes | Yes | Yes | Some |
| CoCoMac | http://cocomac. org/home.asp | Bakker et al., 2012 | Brain regions, connections | No | No | Yes | No | No | No | No |
| Temporal Lobe | http://www. temporal-lobe. com/ | van Strien et al., 2009 | Neurons, connections, brain regions | No | No | Yes | No | No | No | Yes |
| BioMedGT | http://biomedgt.nci. nih.gov/wiki/index. php/Main_Page | Solbrig & Jiang, 2009 | Cancer-related topics | Yes | Some | Yes | Yes | No | Yes | N/A |
| NeuroNames | http://braininfo. rprc.washington. edu/nnont.aspx | Bowden & Dubach, 2003 | Neurons, brain regions | No | No | Yes | No | No | Yes | No |
| NIF STD | http://bioportal. bioontology. org/ontologies/4051 | Bug et al., 2008 | Neurons, other cells, brain regions, connections, proteins & molecules, species, information resources | No | No | Yes | Yes | Yes | No | Yes |
| GeneWiki | http://en.wikipedia. org/wiki/Portal: Gene_Wiki | Good et al., 2011 | Genes, proteins | Yes | Yes | Yes | No | No | Yes | N/A |
| Cognitive Atlas | http://www. cognitiveatlas.org/ | Miller et al., 2010 | Cognitive processes | Yes | Yes | Yes | Some | No | Yes | N/A |
| Channelpedia | http: //channelpedia. epfl.ch/ | Ranjan et al., 2011 | Proteins (ion channels) | No | No | Yes | No | No | Yes | N/A |
| NeuronBank | http://neuronbank. org/index.php? section=1 | Katz et al., 2010 | Neurons | No | Yes | Yes | No | No | Yes | Yes |
| Foundational Model of Anatomy | http://sig.biostr. washington. edu/projects/fm/ | Rosse & Mejino, 2003 | Brain regions, other anatomical features | No | No | Yes | Yes | Yes | No | N/A |
| OBO Foundry | http://www. obofoundry.org/ | Smith et al., 2007 | | No | No | Yes | Yes | Yes | No | Some |
