# Supplementary material for: NeuroLex.org: an online framework for neuroscience knowledge
Source: Front Neuroinform. 2013 Aug 30;7:18. doi: 10.3389/fninf.2013.00018 (PMC3757470; doi:10.3389/fninf.2013.00018)
Supplement: Supplementary file 3 [file DataSheet3.PDF]

Neuron properties

Standard

- Definition
- Synonym
- Related to
- Has role (Principle neuron role, intrinsic neuron role)
- Abbreviation
- Supercategory
- Defining citation
- Publication link
- Id
- PMID
- Organism

Soma Specific Properties

- Cell soma shape (Fusiform, Granule, Horizontal, Multipolar, Oval, Pear-shaped, Pyramidal, Spherical, Mitral)
- Cell soma size (Large soma, Medium soma, small soma)
- Soma location (*Autocomplete from brain regions*)

Dendrite Specific Properties

- Dendrite location (*Autocomplete from brain regions*)
- Spine density on dendrites (Smooth, spiny low density, spiny high density)
- Branching type (unipolar, bipolar, apical/basal, multipolar, stellate)

Axon Specific Properties

- Axon myelination (myelinated, unmyelinated)
- Axon projection laterality (ipsilateral, contralateral, bilateral)
- Origin of axon (soma, soma and sometimes dendrite, dendrite, no axon)
- Location of distant axon arborization (*Autocomplete from brain regions*)
- Location of local axon arborization (*Autocomplete from brain regions*)

Intrinsic Properties

- Neurotransmitter released (*Autocomplete from brain regions*)
- Neurotransmitter receptors (*Autocomplete from brain regions*)
- Molecular constituents (*Autocomplete from brain regions*)
- Firing patterns (Regular spiking, bursting, Regular firing and bursting, Fast-spiking,

Plateau potentials)
- Spontaneous firing patterns (Regular spiking, bursting, Regular firing and bursting)
- Spontaneous firing rate (Rapid firing, medium firing, slow firing)

Brain Region properties

- Definition
- Synonym(s)
- Is part of
- Related To
- Has Role
- Abbreviation
- SuperCategory
- Defining Citation
- Id
- PMID
- Publication Link
- Organism
- NeuronamesID
- Defining Criteria
- Cyto-architecture defining criteria
- Myelo-architecture defining criteria
- Connectivity defining criteria
- Tomography defining criteria
- Topography defining criteria
- Chemoarchitecture defining criteria
- Profile (expression, binding, physiology)
- Coordinates
- Atlas Image
- Brain regions that send axons into this region
- Brain regions that receive axons from this region
- Overlaps
- Parcellation scheme

Resource properties

- Description
- Other name(s)
- Parent organization

- Related To
- Resource Type(s)
- Keywords
- Abbreviation
- SuperCategory
- URL
- Id
- PMID
- Publication Link
- Related disease
- Related application
- Processing
- Availability
- Organism
- CAO Id
- DICOM ID
- Contributor
- Cognitive Atlas Link
